# *PIK3R1*, *HRAS* and *AR* Gene Alterations Associated with Sclerosing Polycystic Adenoma of the Parotid Gland

**DOI:** 10.3390/cimb45020061

**Published:** 2023-01-19

**Authors:** Hisham F. Bahmad, Gina Elhammady, Jennifer M. Gass, Juan C. Paramo, Robert Poppiti, John Alexis

**Affiliations:** 1 The Arkadi M. Rywlin M.D. Department of Pathology and Laboratory Medicine, Mount Sinai Medical Center, Miami Beach, FL 33140, USA; 2 Florida Cancer Specialists & Research Institute, Fort Myers, FL 33916, USA; 3 Department of General Surgery, Mount Sinai Medical Center, Miami Beach, FL 33140, USA; 4 Department of Translational Medicine, Herbert Wertheim College of Medicine, Florida International University, Miami, FL 33199, USA

**Keywords:** sclerosing polycystic adenoma, parotid gland, next generation sequencing, *HRAS*, *AR*, *PIK3R1*

## Abstract

Sclerosing polycystic adenoma (SPA) is a rare neoplasm occurring in the salivary glands, mainly the parotid gland. Although it was originally thought to represent a non-neoplastic process, recent genetic data have proven its monoclonality, supporting its neoplastic origin. We report a case of a 73-year-old woman who presented with left neck swelling and pain. A 3 cm hypoechoic, heterogeneous, solid mass was identified on neck ultrasonography within the left parotid gland. Fine needle aspiration revealed benign acinar cells and lymphocytes. Left partial superficial parotidectomy was performed and a diagnosis of SPA was made. Targeted next-generation sequencing (NGS) revealed three clinically significant alterations in the *PIK3R1*, *HRAS*, and *AR* genes. Alterations in the *PIK3R1* gene have been previously reported in cases of SPA; however, this study is the first to report two novel clinically significant genomic alterations in the *HRAS* and *AR* genes. AR protein expression by immunohistochemistry was strongly and diffusely positive in the neoplastic epithelial cells compared to the adjacent normal salivary gland tissue, which was dead negative for AR. This molecular profile will enhance our understanding of the molecular pathways underlying the development of this tumor. Although this entity was initially thought to be a reactive process, evidence from our case and similar cases strongly support the notion that it is neoplastic due to the presence of specific genetic alterations linked to it.

## 1. Introduction

Sclerosing polycystic adenoma (SPA) is an uncommon entity occurring in the salivary glands, with the majority of cases (70%) reported in the parotid gland and around 60 cases reported worldwide [1]. SPA was first described in 1996 by Smith et al. in a case series of nine patients aged from 12 to 63 years [2]. The age range for SPA is wide (7 to 84 years) with women being affected more than men and a mean presentation during the fifth decade [1,2,3,4,5,6,7,8,9]. The histologic features resemble fibrocystic disease of the breast [10]. In addition, SPA may show foci of apocrine, intraductal proliferation resembling ductal neoplasia of the breast [10]. The exact nature of the disease is still unknown; however, recent evidence strongly suggests it is neoplastic [11].

The gross appearance consists of one or more well-delineated, pale, firm, rubbery nodules embedded within a normal salivary gland. Microscopically, the nodules consist of irregularly distributed, bilayered ducts and acini within a sclerotic stroma. Ductal cells may have vacuolated, foamy, apocrine, and mucous appearances. Acini contain numerous, coarse eosinophilic, periodic acid Schiff (PAS)-positive, intracytoplasmic granules. Genetic studies on SPA have reported X-chromosome inactivation, *PTEN* loss, and *PIK3CA* and *PIK3R1* mutations [3]. Management consists of surgical excision and patients have an excellent prognosis, with recurrence occurring in about 10% of the reported cases [10] and only one case reporting malignant transformation into invasive carcinoma after multiple recurrences over a period of years [9].

In this report, we present a case of a 73-year-old woman who presented to our institution due to left neck swelling and pain. A 3 cm hypoechoic, heterogeneous, circumscribed, solid mass was identified on neck ultrasonography within the left parotid gland. Left partial superficial parotidectomy was performed and a diagnosis of SPA was made based on histopathologic examination. Targeted next-generation sequencing (NGS) was performed showing three clinically significant alterations in the *PIK3R1*, *HRAS*, and *AR* genes. This surgical case report was conducted and reported in accordance with Surgical CAse REports (SCARE) guidelines for reporting case reports.

## 2. Case Presentation

A 73-year-old woman with a medical history of hypertension and thyroid disease presented to our institution with left neck swelling and pain. The patient never smoked and had no known allergies. Family history was not pertinent. Physical examination revealed a 2 cm firm smooth palpable left neck mass. A neck ultrasonography showed a hypoechoic heterogeneous solid mass within the left parotid gland measuring 2.9 × 2.9 × 2.2 cm. A computed tomography (CT) scan of the neck showed a 2.7 cm solid-appearing mass in the left parotid gland. A neoplastic process was favored. Fine needle aspiration revealed clusters of benign acinar cells in a background of scattered lymphocytes, favoring a benign pathologic process.

**Figure 1:**
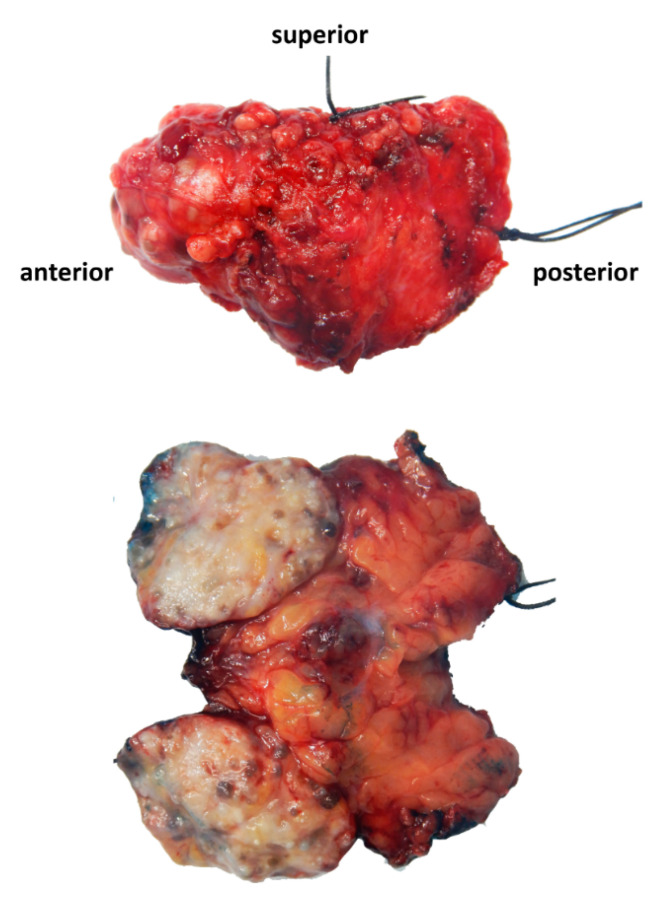
Gross image of the left partial superficial parotidectomy specimen. A 2.8 cm circumscribed and lobulated mass was seen occupying the anterior portion of the parotid gland, with tan, white, focally yellow cut surface and cystic areas on cut section.

**Figure 2:**
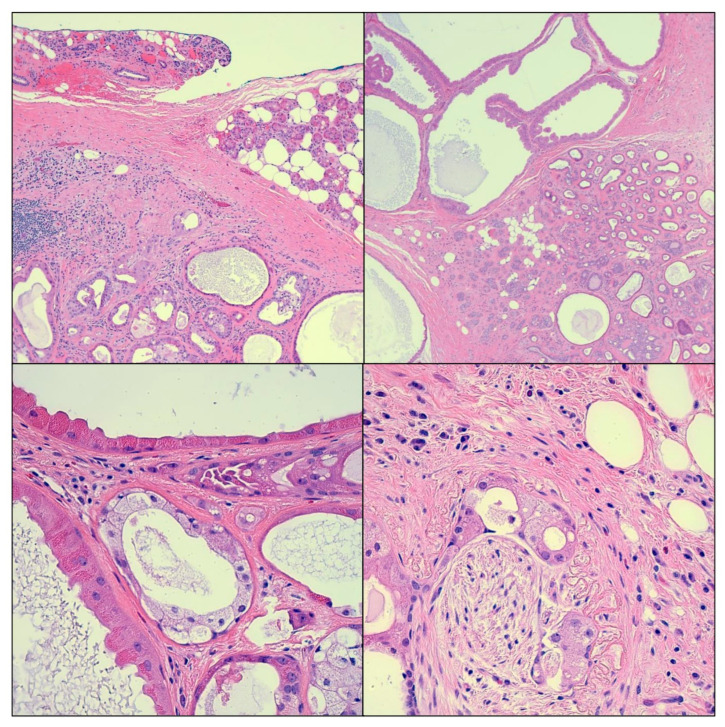
Microscopic images showing proliferation of acini, ducts, and cysts, with intraductal epithelial proliferation. Cysts are lined by cells with granular cytoplasm and apocrine features. Areas of fibrosis and chronic and xanthogranulomatous inflammation are also present. The tumor lacks anaplasia; however, a microscopic focus of perineural entrapment is seen. Images were taken at 50× and 400× magnification.

Left partial superficial parotidectomy with facial nerve dissection and nerve integrity monitoring was performed and the specimen was submitted to Pathology. The surgical specimen weighed 14 g and measured 5.3 cm from superior to inferior, 3.2 cm from anterior to posterior, and 1.6 cm from superficial to deep. It consisted of a 2.8 × 2.3 × 2 cm circumscribed and lobulated mass with a tan, white, focally yellow cut surface and multiple cystic areas (Figure 1). The mass appeared to be abutting the anterior, superficial, and deep margins. Microscopic examination revealed a proliferation of acini, ducts, and cysts. Some ducts exhibited intraductal epithelial proliferation with apocrine metaplasia resembling atypical ductal hyperplasia of the breast. Cysts were lined by eosinophilic cells with brightly eosinophilic cytoplasmic granules (Figure 2). Other findings included fibrosis, chronic, and xanthogranulomatous inflammation. The tumor appeared circumscribed and lacked anaplasia; however, a microscopic focus of perineural entrapment was also seen. Based on the pathologic findings, a diagnosis of sclerosing polycystic adenoma was made. The case was sent out in consultation, wherein there was an agreement with the diagnosis of sclerosing polycystic adenoma.

Targeted NGS was performed, and the results are summarized in Table 1. The Illumina TruSight™ Oncology 500 (TSO500) targeted hybrid-capture kit was used to target known disease-associated regions in DNA (523 genes) and RNA (55 genes). The targeted regions were sequenced using the Illumina NextSeq^®^ 550Dx System with 101bp paired-end reads. The DNA and RNA data were analyzed using the Illumina Software TSO500 v2.0.0, a bioinformatics pipeline, and a customized analysis pipeline from PierianDx. The DNA sequence was aligned and compared to the human reference genome GRCh37p.13 (GRCh37/h19). The Clinical Genomics Workspace software platform from PierianDx was used to call, filter, and analyze variants found in the patient sample. TSO500 can detect multiple classes of variants, including single-nucleotide variants, multi-nucleotide variants (<3bp), small insertions (1-18bp) and deletions (1-27bp), copy-number variants, fusions, and splice variants. The assay quantitatively detects microsatellite instability (MSI) and tumor mutational burden (TMB).

Among its limitations, certain types of genetic alterations including large deletions and duplications, complex rearrangements, and repeat expansions may not be detected by the current analysis. A pathogenic change may not be detected if it is located outside of the targeted regions. The following untargeted exons have been identified: HIST2H3A NM_001005464.2 exon 1, HIST2H3C NM_021059.2 exon 1, MYB NM_001130173.1 exon 1, PAX8 NM_003466.3 exon 8, DPK1 NM_002613.4 exon 10, PDPK1 NM_002613.4 exon 3, PDPK1 NM_002613.4 exon 8, PDPK1 NM_002613.4 exon 6, PDPK1 NM_002613.4 exon 4, PDPK1 NM_002613.4 exon 5, PDPK1 NM_002613.4 exon 9, RANBP2 NM_006267.4 exon 13, RANBP2 NM_006267.4 exon 8, REL NM_002908.2 exon 9, RICTOR NM_152756.3 exon 19, and SUZ12 NM_015355.2 exon 3. Additionally, all small variant calls in the *HLA-A*, *KMT2B*, *KMT2C*, and *KMT2D* genes are filtered out due to potential mis-mapping as a result of sequence homology with other genomic regions. It is possible that pathogenic variants may not be reported by one or more of the tools because of the parameters used. However, tool parameters were optimized to maximize specificity and sensitivity.

As a result, three clinically significant alterations in the *PIK3R1*, *HRAS*, and *AR* genes were identified. No loss of *PTEN* was found. Specifically, the lesion harbored a missense hotspot alteration, c.182A>G (p.Q61R), in the *HRAS* gene, a missense alteration, c.1690A>G (p.N564D), in the *PIK3R1* gene, and a splice site alteration, AR-V7, in the *AR* gene. In addition, six genomic variants of uncertain significance were identified, including *DIS3* (p.T899I; NM_014953.3:c.2696C>T), *ETV4* (p.K6*; NM_001079675.2: c.16A>T), *HGF* (p.M414T; NM_000601.4: c.1241T>C), *IRS2* (p.P807L; NM_003749.2: c.2420C>T), *PREX2* (p.D1100E; NM_024870.2: c.3300C>G), and *SPEN* (p.P542S; NM_015001.2: c.1624C>T). Microsatellite instability (MSI) and tumor mutational burden (TMB) scores were also tested, showing a stable MSI score of 4.1% (≤20% is considered stable) and a low TMB score of 4.8 muts/Mb (a high TMB score is defined as ≥10 Mutb/Mb).

**Figure 3:**
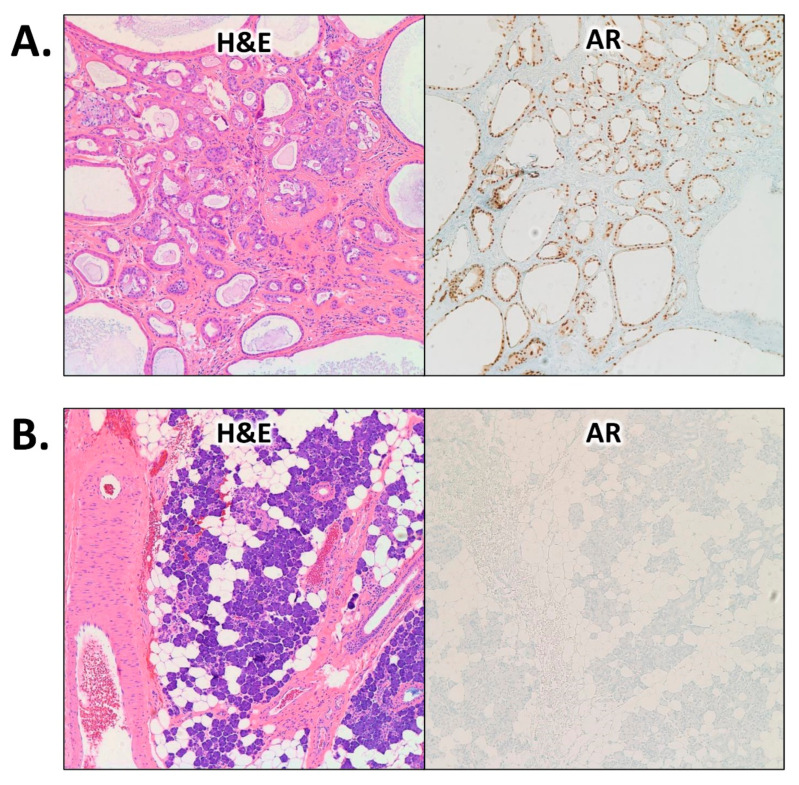
Microscopic images showing positive immunohistochemical protein expression of AR in the neoplastic epithelial cells (diffuse and strong nuclear staining) in (**A**) compared to the adjacent normal salivary gland tissue that was dead negative for AR in (**B**). Images were taken at 100× magnification. Abbreviations: H&E: hematoxylin and eosin; AR: androgen receptor.

We performed immunohistochemical (IHC) staining to assess the protein expression of AR using an automated Benchmark ULTRA staining platform (Ventana Medical Systems, Tucson, AZ, United States). Interestingly, there was strong and diffuse AR expression in the neoplastic epithelial cells, whereas the adjacent normal salivary gland tissue was dead negative for AR and served as an internal negative control (Figure 3).

Post-operatively, the patient recovered well with no clinical signs or symptoms of hypocalcemia (calcium level at 8.2 mg/dL post-operatively; reference range: 8.5–10.1 mg/dL) or hematoma formation. The patient was discharged the next day. On follow up after 1 year, the patient appeared to be recovering satisfactorily. The surgical incision was well-healed. Accordingly, no further therapy was warranted at this time and routine follow up was recommended.

## 3. Discussion

Sclerosing polycystic adenoma (SPA) consists of a circumscribed, lobular proliferation of acini and ducts with granular, vacuolated, or apocrine cellular features. Acini contain coarse red zymogen granules embedded in a fibrotic stroma, whereas the ductal elements are proliferative, creating a resemblance to low-grade intraductal carcinoma (“ductal carcinoma in situ”). Although SPA has histologic features that resemble fibrocystic changes of the breast, the admixture of acini, ducts, and sclerotic stroma present in this lesion makes it unique, unlike other well-established salivary gland neoplasms. In the 2017 World Health Organization Classification of Head and Neck Tumors, SPA was classified as a reactive non-neoplastic lesion [12]. Nevertheless, enough data are being published confirming its monoclonal (and hence neoplastic) nature, including our case [3,10]. The first study to shed light on this was conducted by Skálová et al. in 2006. In this study, the human androgen receptor assay (HUMARA) was utilized, exhibiting the presence of X-chromosome inactivation in six cases of SPA and providing strong evidence of monoclonality [3].

In our patient, a total of 523 genes were subjected to targeted NGS analysis. Splice site alteration, AR-V7, was identified in the androgen receptor (*AR*) gene. This alteration leads to the loss of the ligand-binding domain of the canonical *AR* transcript [13], causing constitutive activation of *AR* [14,15]. The latter belongs to the nuclear hormone receptor family, which acts as a transcription factor. Overexpression of *AR* is common in prostate cancer (PCa) [16] and is associated with resistance to anti-androgen therapy [17,18]. For instance, a study by Antonarakis et al. showed that circulating tumor cells harboring AR-V7 alterations from patients with advanced PCa are likely to be resistant to enzalutamide and abiraterone [17]. Interestingly, comprehensive molecular and expression analysis of the *AR* gene in 35 tumor specimens and cell lines derived from salivary duct carcinoma revealed AR overexpression in 70% of the cases [19]. This suggests that overactive AR signaling, which is an important oncogenic driver in PCa and possibly plays a role in salivary duct carcinoma, could be implicated in the pathogenesis of SPA [20].

In our case, a missense hotspot alteration, c.182A>G (p.Q61R), was also identified in the *HRAS* proto-oncogene. This alteration results in decreased HRAS GTPase activity [21]. *HRAS* is a member of the small GTPase family that, upon activation by growth factors, stimulates multiple downstream pathways such as RAF and PI3K to promote cell proliferation and survival [22,23]. Activating mutations in this gene have been commonly described in various tumors, including skin, head and neck, thyroid, kidney, and bladder cancers [24]. In salivary gland malignancies, *HRAS* mutations represent more than 90% of all *RAS* mutations, and they mainly occur in epithelial-myoepithelial carcinomas and salivary duct carcinomas [25].

Lastly, a missense alteration, c.1690A>G (p.N564D), was identified in the *PIK3R1* gene. This alteration confers a loss of function to the PIK3R1 protein, leading to constitutive PI3K signaling, phosphorylation of AKT, and increased tumor growth, as demonstrated in animal models [26,27,28,29]. A study by Bishop et al. in 2020 including four patients with SPA showed that all cases tested with targeted NGS harbored mutations in genes that are members of the PI3K cell cycle regulation pathway, including *PTEN*, *PIKCA*, and *PIK3R1* [10], which is consistent with our patient. Genetic mutations within the PI3K pathway are found in most tumors. The PI3K/AKT/mTOR pathway controls cell proliferation and survival and is an important therapeutic target in many cancer types [30]. Interestingly, a role for PI3K signaling had been defined in salivary duct carcinomas, rendering this pathway a target for personalized therapy [30,31,32,33]. Moreover, PI3K pathway alterations have been implicated in intraductal carcinomas of the salivary gland (apocrine variant) [34].

Due to the histologic features of this tumor, the main differential diagnosis of SPA includes chronic sclerosing sialadenitis (which exhibits dense lymphoplasmacytic infiltrate with lymphoid follicles) and intraductal carcinoma (a more complex cystic pattern with nuclear atypia). In fact, since both SPA and apocrine intraductal carcinoma harbor similar genetic aberrations, we hypothesize that those two entities may be closely related, and that SPA may indeed be a precursor lesion to apocrine intraductal carcinoma. Other neoplasms of the salivary glands have not been shown to harbor *PTEN* mutations, including pleomorphic adenoma, acinic cell carcinoma, and mucoepidermoid carcinoma. Nonetheless, genomic alterations in *HRAS* and *PIK3R1*, which we found in our case, have also been described in salivary duct carcinomas, implying a mutual genetic background among neoplasms occurring in the salivary glands. However, each behaves differently in a benign or malignant fashion. In SPA, recurrence has been reported only in around 10% of cases, and this is attributed to either multifocality or incomplete excision of the lesion [5]. Furthermore, only one case of SPA had been reported to undergo malignant transformation after three recurrences over a span of 32 years [9].

## 4. Conclusions

In conclusion, sclerosing polycystic adenoma (SPA) is a rare neoplasm occurring in the salivary glands, mainly the parotid glands, with around 60 cases reported worldwide. Genetic studies on patients with SPA have reported X-chromosome inactivation, *PTEN* loss, and *PIK3CA* and *PIK3R1* mutations. We report, for the first time, two novel clinically significant genomic alterations in the *HRAS* and *AR* genes in a SPA of a 73-year-old woman, not including *PIK3R1* gene alteration, which has been already reported. This molecular profile will enhance our understanding of the molecular pathways underlying the development of this benign neoplasm.

## Figures and Tables

**Table 1:** Next-generation sequencing results for the sclerosing polycystic adenoma.

Gene	Alteration	Reference Sequence	Nucleotide Change	VAF
*AR*	AR-V7	NM_000044.3	--	--
*HRAS*	p.Q61R	NM_005343.2	c.182A>G	0.7%
*PIK3R1*	p.N564D	NM_181523.2	c.1690A>G	23.9%

Abbreviations: *AR*: androgen receptor; *HRAS*: HRas proto-oncogene; *PIK3R1*: phosphoinositide-3-kinase regulatory subunit 1; VAF: variant allele frequency.

## Data Availability

Not applicable.

## References

[1] Gnepp D.R. (2014). Salivary gland tumor “wishes” to add to the next WHO Tumor Classification: Sclerosing polycystic adenosis, mammary analogue secretory carcinoma, cribriform adenocarcinoma of the tongue and other sites, and mucinous variant of myoepithelioma. Head Neck Pathol..

[2] Smith B.C., Ellis G.L., Slater L.J., Foss R.D. (1996). Sclerosing polycystic adenosis of major salivary glands. A clinicopathologic analysis of nine cases. Am. J. Surg. Pathol..

[3] Skálová A., Gnepp D.R., Simpson R.H., Lewis J.E., Janssen D., Sima R., Vanecek T., Di Palma S., Michal M. (2006). Clonal nature of sclerosing polycystic adenosis of salivary glands demonstrated by using the polymorphism of the human androgen receptor (HUMARA) locus as a marker. Am. J. Surg. Pathol..

[4] Skálová A., Michal M., Simpson R.H., Stárek I., Prádná J., Pfaltz M. (2002). Sclerosing polycystic adenosis of parotid gland with dysplasia and ductal carcinoma in situ. Report of three cases with immunohistochemical and ultrastructural examination. Virchows Arch..

[5] Gnepp D.R., Wang L.J., Brandwein-Gensler M., Slootweg P., Gill M., Hille J. (2006). Sclerosing polycystic adenosis of the salivary gland: A report of 16 cases. Am. J. Surg. Pathol..

[6] Petersson F. (2013). Sclerosing polycystic adenosis of salivary glands: A review with some emphasis on intraductal epithelial proliferations. Head Neck Pathol..

[7] Mokhtari S., Atarbashi Moghadam S., Mirafsharieh A. (2014). Sclerosing polycystic adenosis of the retromolar pad area: A case report. Case Rep. Pathol..

[8] Su A., Bhuta S.M., Berke G.S., Lai C.K. (2013). A unique case of sclerosing polycystic adenosis of the sinonasal tract. Hum. Pathol..

[9] Canas Marques R., Félix A. (2014). Invasive carcinoma arising from sclerosing polycystic adenosis of the salivary gland. Virchows Arch..

[10] Bishop J.A., Gagan J., Baumhoer D., McLean-Holden A.L., Oliai B.R., Couce M., Thompson L.D.R. (2020). Sclerosing Polycystic “Adenosis” of Salivary Glands: A Neoplasm Characterized by PI3K Pathway Alterations More Correctly Named Sclerosing Polycystic Adenoma. Head Neck Pathol..

[11] Skálová A., Gnepp D.R., Lewis J.S., Hunt J.L., Bishop J.A., Hellquist H., Rinaldo A., Vander Poorten V., Ferlito A. (2017). Newly Described Entities in Salivary Gland Pathology. Am. J. Surg. Pathol..

[12] El-Naggar A.K., Chan J.K., Grandis J.R. (2017). WHO Classification of Head and Neck Tumours.

[13] Nakazawa M., Antonarakis E.S., Luo J. (2014). Androgen receptor splice variants in the era of enzalutamide and abiraterone. Horm. Cancer.

[14] Hu R., Dunn T.A., Wei S., Isharwal S., Veltri R.W., Humphreys E., Han M., Partin A.W., Vessella R.L., Isaacs W.B. (2009). Ligand-independent androgen receptor variants derived from splicing of cryptic exons signify hormone-refractory prostate cancer. Cancer Res..

[15] Watson P.A., Chen Y.F., Balbas M.D., Wongvipat J., Socci N.D., Viale A., Kim K., Sawyers C.L. (2010). Constitutively active androgen receptor splice variants expressed in castration-resistant prostate cancer require full-length androgen receptor. Proc. Natl. Acad. Sci. USA.

[16] Culig Z., Bartsch G. (2006). Androgen axis in prostate cancer. J. Cell. Biochem..

[17] Antonarakis E.S., Lu C., Wang H., Luber B., Nakazawa M., Roeser J.C., Chen Y., Mohammad T.A., Chen Y., Fedor H.L. (2014). AR-V7 and resistance to enzalutamide and abiraterone in prostate cancer. N. Engl. J. Med..

[18] Boudadi K., Antonarakis E.S. (2016). Resistance to Novel Antiandrogen Therapies in Metastatic Castration-Resistant Prostate Cancer. Clin. Med. Insights Oncol..

[19] Mitani Y., Rao P.H., Maity S.N., Lee Y.C., Ferrarotto R., Post J.C., Licitra L., Lippman S.M., Kies M.S., Weber R.S. (2014). Alterations associated with androgen receptor gene activation in salivary duct carcinoma of both sexes: Potential therapeutic ramifications. Clin. Cancer Res..

[20] Dalin M.G., Watson P.A., Ho A.L., Morris L.G. (2017). Androgen Receptor Signaling in Salivary Gland Cancer. Cancers.

[21] Der C.J., Finkel T., Cooper G.M. (1986). Biological and biochemical properties of human rasH genes mutated at codon 61. Cell.

[22] Fernández-Medarde A., Santos E. (2011). Ras in cancer and developmental diseases. Genes Cancer.

[23] Shu L., Wang D., Saba N.F., Chen Z.G. (2020). A Historic Perspective and Overview of H-Ras Structure, Oncogenicity, and Targeting. Mol. Cancer Ther..

[24] Khan A.Q., Kuttikrishnan S., Siveen K.S., Prabhu K.S., Shanmugakonar M., Al-Naemi H.A., Haris M., Dermime S., Uddin S. (2019). RAS-mediated oncogenic signaling pathways in human malignancies. Semin. Cancer Biol..

[25] Grünewald I., Vollbrecht C., Meinrath J., Meyer M.F., Heukamp L.C., Drebber U., Quaas A., Beutner D., Hüttenbrink K.B., Wardelmann E. (2015). Targeted next generation sequencing of parotid gland cancer uncovers genetic heterogeneity. Oncotarget.

[26] Ross R.L., Burns J.E., Taylor C.F., Mellor P., Anderson D.H., Knowles M.A. (2013). Identification of mutations in distinct regions of p85 alpha in urothelial cancer. PLoS ONE.

[27] Jaiswal B.S., Janakiraman V., Kljavin N.M., Chaudhuri S., Stern H.M., Wang W., Kan Z., Dbouk H.A., Peters B.A., Waring P. (2009). Somatic mutations in p85alpha promote tumorigenesis through class IA PI3K activation. Cancer Cell.

[28] Ng P.K., Li J., Jeong K.J., Shao S., Chen H., Tsang Y.H., Sengupta S., Wang Z., Bhavana V.H., Tran R. (2018). Systematic Functional Annotation of Somatic Mutations in Cancer. Cancer Cell.

[29] Chen L., Yang L., Yao L., Kuang X.Y., Zuo W.J., Li S., Qiao F., Liu Y.R., Cao Z.G., Zhou S.L. (2018). Characterization of PIK3CA and PIK3R1 somatic mutations in Chinese breast cancer patients. Nat. Commun..

[30] Ettl T., Schwarz-Furlan S., Haubner F., Müller S., Zenk J., Gosau M., Reichert T.E., Zeitler K. (2012). The PI3K/AKT/mTOR signalling pathway is active in salivary gland cancer and implies different functions and prognoses depending on cell localisation. Oral Oncol..

[31] Saintigny P., Mitani Y., Pytynia K.B., Ferrarotto R., Roberts D.B., Weber R.S., Kies M.S., Maity S.N., Lin S.H., El-Naggar A.K. (2018). Frequent PTEN loss and differential HER2/PI3K signaling pathway alterations in salivary duct carcinoma: Implications for targeted therapy. Cancer.

[32] Griffith C.C., Seethala R.R., Luvison A., Miller M., Chiosea S.I. (2013). PIK3CA mutations and PTEN loss in salivary duct carcinomas. Am. J. Surg. Pathol..

[33] Chiosea S.I., Williams L., Griffith C.C., Thompson L.D., Weinreb I., Bauman J.E., Luvison A., Roy S., Seethala R.R., Nikiforova M.N. (2015). Molecular characterization of apocrine salivary duct carcinoma. Am. J. Surg. Pathol..

[34] Weinreb I., Bishop J.A., Chiosea S.I., Seethala R.R., Perez-Ordonez B., Zhang L., Sung Y.S., Chen C.L., Assaad A., Oliai B.R. (2018). Recurrent RET Gene Rearrangements in Intraductal Carcinomas of Salivary Gland. Am. J. Surg. Pathol..

